# Incidental gallbladder cancer: what is the prevalence and how do we perform cholecystectomy for presumably benign biliary disease?

**DOI:** 10.1590/0100-6991e-20223417-en

**Published:** 2022-12-09

**Authors:** DIEGO ARLEY GOMES DA SILVA, OLGA LANUSA LEITE VELOSO, MATHEUS SOUTO PERAZZO VALADARES, RODRIGO SOARES DA COSTA, MARIANA GALINDO SILVEIRA, FERNANDA COSTA DE CARVALHO, MARCELO GONÇALVES SOUSA

**Affiliations:** 1 - Universidade Federal da Paraíba, Departamento de Cirurgia - João Pessoa - PB - Brasil; 2 - Instituto do Coração do Hospital das Clínicas da FMUSP, Disciplina de Cirurgia Torácica - São Paulo - SP - Brasil

**Keywords:** Gallstones, Gallbladder Diseases, Gallbladder Neoplasms, Cálculos Biliares, Doenças da Vesícula Biliar, Neoplasias da Vesícula Biliar

## Abstract

**Objective:**

to determine the prevalence of incidental gallbladder cancer (IGBC) in cholecystectomies performed in a tertiary public hospital and to describe technical and epidemiological aspects of performing cholecystectomies for presumably benign disease.

**Method:**

descriptive, retrospective observational study, based on analysis of medical records of patients undergoing cholecystectomy with preoperative hypothesis of benign disease between January 2018 and January 2022.

**Results:**

prevalence of gallbladder adenocarcinoma in our sample was 0.16%, similar to data in the literature. Technical aspects during cholecystectomy were also described with a frequency similar to that found in the literature.

**Conclusion:**

despite a rare disease, IGBC is relevant in the routine of the General Surgeon. Its diagnosis, staging and treatment directly affect the prognosis. Technical aspects during cholecystectomy are not always remembered by surgeons and can interfere with the prognosis and subsequent treatment of the patient.

## RESUMO

**Objetivos:**

determinar a prevalência de câncer incidental de vesícula biliar (CIVB) em colecistectomias realizadas em um hospital público terciário e descrever aspectos técnicos e epidemiológicos na realização de colecistectomias por doença presumidamente benigna.

**Método:**

estudo observacional descritivo, retrospectivo, a partir da análise de prontuários de pacientes submetidos à colecistectomia com hipótese pré-operatória de doença benigna entre janeiro de 2018 e janeiro de 2022.

**Resultados:**

a prevalência de adenocarcinoma de vesícula biliar na amostra avaliada foi de 0,16%, semelhante aos dados da literatura mundial. Os aspectos técnicos durante a realização da colecistectomia também foram descritos com frequência semelhante ao encontrado na literatura.

**Conclusão:**

o CIVB é uma doença rara mas de importância relevante na rotina do Cirurgião Geral. Seu diagnóstico, estadiamento e tratamento implicam diretamente no prognóstico. Aspectos técnicos durante a realização da colecistectomia nem sempre são lembrados pelos cirurgiões e podem interferir no prognóstico e tratamento subsequente do paciente.

## INTRODUCTION

Cholelithiasis is the most prevalent disease of the biliary tree, responsible for most elective surgeries performed annually^1^ ^,^ ^2^. It is mainly characterized by abdominal complaints and its leading treatment is laparoscopic cholecystectomy^3^ ^,^ ^4^, currently recommended early in the absence of contraindications, to prevent disease-related complications and symptoms’ recurrence^5^.

Gallbladder carcinoma is a rare malignant neoplasm, with high lethality and rapid progression of symptoms, variable incidence and prevalence, corresponding to the biliary tract tumor with the lowest survival rate at diagnosis^3^ ^,^ ^6^ ^-^ ^8^ and whose most prevalent risk factor is cholelithiasis^9^.

More recently, it has been incidentally diagnosed as a finding after laparoscopic cholecystectomies^6^. The management of incidental gallbladder cancer was recently established in the Brazilian Consensus of the disease^10^.

The disease’s prognosis is variable, according to the stage at the time of diagnosis, and its surgical treatment (re-resection) is indicated when there is no distant disease, which may include liver resections, retroperitoneal lymphadenectomy, and resection of extrahepatic bile duct or other organs, based on histopathological findings and staging imaging tests^11^.

Some technical aspects in cholecystectomies must be considered in view of the incidental diagnosis of the neoplasm. Injuries to the gallbladder wall and bile leakage can interfere with staging and change the prognosis, and should be avoided^12^.

## GOALS

The objectives of this research are to determine the prevalence of incidental gallbladder cancer (IGBC) in cholecystectomies performed between 2018 and 2021 at the Lauro Wanderley University Hospital (HULW), at the Federal University of Paraíba, in João Pessoa, State of Paraíba, Brazil, and to describe its epidemiological aspects and operative technique used to perform cholecystectomies at the institution between August 2021 and January 2022.

## METHODS

This is a descriptive, retrospective, observational study, using a non-probabilistic, convenience sample, which included all medical records of patients undergoing cholecystectomy with indication for gallstones and/or their complications in a highly complex public hospital in João Pessoa, Paraíba, between January 2018 and January 2022.

We excluded all patients who underwent cholecystectomy for other pathologies or who had an established or presumed diagnosis of gallbladder neoplasia or periampullary tumors.

In the epidemiological and surgical evaluation, we included patients who underwent laparoscopic or conventional cholecystectomy in the HULW with a preoperative diagnosis of benign biliary disease, admitted to the institution between August 2021 and January 2022.

To calculate the annual prevalence of gallbladder incidental malignancy, we evaluated pathological anatomy reports performed at the HULW in cholecystectomies (open or laparoscopic) between January 2018 and December 2021.

**Figure 1:**
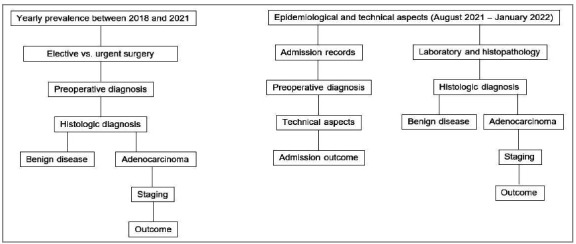
Flowchart for data collection and systematization.

The data collected in the standardized record from the established flowchart (Figure 1) were tabulated using the descriptive statistics tool of the Microsoft Excel software, for the calculation of frequencies, percentages, means, and standard deviations (SD), and subsequently arranged in tables.

The project was submitted for evaluation by the Ethics in Research Committee of the institution, approved under CAAE nº 55395922.2.0000.5183, in accordance with Resolution nº 466/12 of the National Health Council.

## RESULTS

We analyzed 642 medical records of patients who underwent cholecystectomies at the HULW between 2018 and 2021. After applying the inclusion and exclusion criteria, we selected 618 patients, 139 (22.5%) male and 479 (77.5%) female. We excluded 24 records in which cholecystectomy was performed as part of procedures in patients with a preoperative clinical and/or histopathological diagnosis of gastric, duodenal, pancreatic, or main bile duct cancer.

The mean age was 43.9 years (±17.78 SD). Of the 618 surgeries performed for presumably benign biliary disease, 596 (96.4%) were scheduled electively and 22 (3.6%) were operated on an urgent basis. The histological diagnosis was made available on average 14.9 days (±8.66) after the surgical procedure.

Table 1 presents the histopathological diagnoses in the evaluated sample. The prevalence of malignant lesions or precursors of gallbladder cancer as an incidental histopathological finding was 0.08% per year in the evaluated sample. Of the 618 surgical specimens, there was lymph node representation (pericistic lymph node, 12c, or Mascagni lymph node) in 59 (9.5%).

**Table 1:** Anatomopathological diagnosis of patients undergoing cholecystectomy at the Lauro Wanderley University Hospital between 2018 and 2021 (n=618).

Diagnosis	Frequency (%)
Gallbladder adenocarcinoma	1 (0.16%)
Acute cholecystitis	3 (0.48%)
Chronic cholecystitis	571 (92.39%)
Chronic cholecystitis acute crisis	38 (6.15%)
Intestinal metaplasia	1 (0.16%)
Low grade dysplasia	2 (0.32%)
Low-grade biliary intraepithelial neoplasia	1 (0.16%)

Chart 1 describes the positive case for gallbladder adenocarcinoma identified in the sample.

**Chart 1:** Positive case for IGBC in the sample between 2018 and 2021.

	Patient 1
Sex	Male
Age	65 years old
Surgery	Laparoscopic cholecystectomy
Histological diagnosis	Gallbladder adenocarcinoma, ulcerated, biliary type, moderately differentiated. Compromised radial (liver bed) and gallbladder neck surgical margins
Postoperative staging	pT3 pNx pMx
Conduct	Re-resection with lymphadenectomy and resection of the port-sites
Complications	Incisional hernia and umbilical tumor recurrence.
Outcome	Minimum overall survival of 18 months.

We included 45 patients in the group of patients submitted to cholecystectomy presumably due to benign biliary disease between August 2021 and January 2022. We excluded two patients, who had undergone pancreaticoduodenectomy due to suspected or confirmed periampullary neoplasia.

There was a predominance of females (36 patients, 80%), and the mean body mass index (BMI) was 29.0kg/m 2 (± 6.58). The mean length of hospital stay was 6.13 days (± 17.38, range 1-113), and 75.6% of the patients were hospitalized for less than or equal to 2 days.

The most reported comorbidity was high blood pressure (37.8%), followed by obesity (31.1%); 42 patients (93.3%) underwent laparoscopic cholecystectomy, while three underwent open surgery. There were no cases of conversion from laparoscopy to laparotomy in the sample.

Resident physicians in the 2^nd^ (73.3%) and 3^rd^ (15.6%) years of the institutional program of Medical Residency in General Surgery, affiliated with the hospital unit, were the main surgeons, under the supervision and participation of the assistant surgeon responsible for the procedure. From the surgical operative reports present in the medical records, we verified that there was gallbladder perforation in 13 cases (28.9%), while there was no violation of the gallbladder in 30 (66.7%), and such information was absent in 2 records (4.4%).

Table 2 describes the technical aspects of the 42 laparoscopic cholecystectomies performed at the HULW between August 2021 and January 2022. An intracavitary drain was not used in the evaluated cholecystectomies.

**Table 2:** Technical aspects in laparoscopic cholecystectomies at the Lauro Wanderley University Hospital between August 2021 and January 2022 (n=42).

Technical aspect	Frequency (%)
Pneumoperitoneum	
Open technique (Hasson)	37 (82.2%)
Veress Needle	5 (11.1%)
Uninformed	0 (0.0%)
Dissection of the cystic duct and cystic artery mostly with:	
Dissector forceps (or Maryland)	12 (29.3%)
Monopolar power (Hook clamp)	29 (70.7%)
Uninformed	1
Dissection of the vesicular bed	
Dissector forceps (or Maryland)	12 (2.4%)
Monopolar power (Hook clamp)	40 (97.6%)
Uninformed	1
Use of bag or glove to remove the gallbladder	
Yes	24 (58.5%)
No	17 (41.5%)
Uninformed	1
Specimen retrieval site	
Epigastric portal	10 (25.0%)
Umbilical portal	30 (75.0%)
Uninformed	2
Emptying of pneumoperitoneum	
With trocar	28 (71.8%)
Without trocar	10 (25.6%)
Not performed	1 (2.6%)
Uninformed	1
Opening and inspection of the specimen by the surgeon	
Yes	11 (28.2%)
No	28 (71.8%)
Uninformed	3

All 45 patients in the sample were discharged from the hospital, with three complications recorded, without the need for surgical reintervention, according to the Clavien-Dindo scale (Table 3).

**Table 3:** Postoperative complications in cholecystectomies performed at the HULW between August 2021 and January 2022 (n=45).

Clavien-Dindo scale for postoperative complications	Frequency (%)
Grade I	1 (2.2%)
Intracavitary collections without need for intervention	1
Grade II	2 (4.4%)
Pneumonia associated with mechanical ventilation	1
Intraperitoneal hematoma requiring blood transfusion	1
Grade III	0 (0.0%)
Grade IV	0 (0.0%)
Grade V	0 (0.0%)

## DISCUSSION

The present research found a prevalence of 0.16% of incidental gallbladder adenocarcinoma in cholecystectomies performed for a presumably benign disease, data corroborated by other similar studies, which show a prevalence ranging from 0.14% to 1.07% (Table 4).

**Table 4:** Prevalence of incidental gallbladder cancer in other studies.

	Study location	Sample	Frequency
Present study	Brazil	618	1 (0.16%)
Sujata, S^13^	India	622	6 (0.96%)
Alabi, Arvind^3^	UK	1,473	2 (0.14%)
Jha, Sharma^9^	India	4,800	20 (0.41%)
Martins-Filho, Batista^8^	Brazil	2,008	10 (0.49%)
Muszynska, Lundgren^12^	Sweden	36,555	215 (0.59%)
Ocon, Vincent^14^	Spain	372	4 (1.07%)
Wu, Li^15^	China	11,589	26 (0.22%)
Tian, Ji^16^	China	7,582	69 (0.91%)

In face of the diagnosis of incidental gallbladder cancer, it is necessary to perform an adequate staging with imaging examination (computed tomography and/or magnetic resonance imaging) and detailed histopathological analysis, which includes, in addition to lymph node involvement, the depth of invasion (T stage), margin of the cystic duct, and perineural and vascular invasion^17^. Other prognostic factors are the degree of tumor differentiation, extent of resection, bile leakage, and type of surgery^16^.

Proper assessment of lymph node status is a fundamental part of the surgical management of patients with gallbladder cancer^18^. In our work, there was lymph node representation in 9.5% of the histopathology specimens evaluated. We found no studies citing the prevalence of lymph node sampling in routine cholecystectomies. Misra, Chaturvedi^19^ points out that, in patients whose neoplasm diagnosis is performed only in the histopathological analysis of the surgical specimen, the management of incidental cancer would take place from the T stage, since generally there is no information about lymph node involvement.

Two Japanese studies in patients undergoing surgical resection with curative intent identified that the cystic (12c), pericholedocian (12b), and posterosuperior peripancreatic (13a) lymph nodes were the initial and, therefore, more prevalent sites of lymphatic metastasis, suggesting that the risk of pN1 pathological staging is 0% when sampling from lymph nodes 12c and 12b is negative for malignancy^20^ ^,^ ^21^.

The mean length of stay of patients undergoing cholecystectomy in our sample was high, probably due to the inclusion of records of patients with long hospitalizations for other clinical diseases and who manifested biliary pathology during hospitalization, motivating surgery on an urgent basis or scheduled within the same hospital stay. An analysis of 985 patients who underwent laparoscopic cholecystectomy between May 2006 and February 2015 in a tertiary hospital in Italy suggested that prolonged hospital stay (defined as greater than 2 days) is not related to the surgical procedure, but to the patient’s comorbidities^22^.

Regarding inflation of the pneumoperitoneum, in our study there was a predominance of the open technique (Hasson or its modifications) compared with the closed one, with a Veress needle. There were no complications related to either technique. There are several studies comparing both techniques regarding the incidence of major and minor complications, the time required to establish pneumoperitoneum, and the technique’s safety, with divergent results favoring one or the other^23^ ^-^ ^25^.

Iatrogenic perforation of the gallbladder with intracavitary bile leakage was present in 28.9% of cases in our sample, at a rate similar to that described in the literature, between 10 and 37%^26^ ^,^ ^27^. This finding is related to the increase in operative time, use of drains^26^, and length of hospital stay, but with no impact on the risk of surgical site infection or postoperative collections^27^.

As for incidental gallbladder cancer, bile extravasation may be associated with incomplete resections and systemic recurrences, as malignant cells may implant from the extravasated content^16^, with a worse prognosis^28^, and a greater probability of peritoneal carcinomatosis, less chance of radical resection, and new surgery with R0 margins, in addition to a shorter disease-free survival time^29^.

There was resection of the laparoscopic port-sites in the patient identified with incidental gallbladder adenocarcinoma, with subsequent reoperation due to incisional hernia and recurrence at the port-sites. Most (58.5%) of the laparoscopic cholecystectomies in our sample used a plastic or latex bag to remove the surgical specimen through the portals. The Brazilian Consensus recommends the routine use of plastic bags for gallbladder removal^10^. In cases where perforation has already occurred intraoperatively, there is no benefit in removing the gallbladder in collection bags^28^.

The guidelines of the European Society of Medical Oncology (ESMO) recommend that the laparoscopic port-sites should be resected in the IGBC when a plastic bag was not used in the removal of the gallbladder or there was bile leakage in the first surgery^17^, while the Brazilian Consensus does not routinely recommend the use of this procedure due to the high incidence of incisional hernia and the reduced oncological benefit^10^.

Opening and inspection of the specimen by the main surgeon did not routinely occur in the surgeries evaluated in our study. According to Tian, Ji^16^, all surgical specimens of the gallbladder must be opened and carefully examined during laparoscopic cholecystectomy, with indication of immediate frozen section biopsy in case of identification of any suspicious lesions, recommendation corroborated by the Brazilian Consensus on IGBC^10^.

The emptying of the pneumoperitoneum at the end of the surgical procedure was performed with at least one trocar positioned in the peritoneal cavity in most of the surgeries described in our study. Animal experimental models from the end of the 20^th^ century suggest the existence of a “Chimney Effect”, in which the circumferential leakage of gas around a trocar could accelerate the infiltration of peritoneal fluid containing aerosolized tumor at the portal site, relating to the activity of that surgical site^30^.

The Brazilian Consensus on IGBC considers that the implantation of tumor cells in the portals’ sites can occur by direct (mechanical factors) and indirect mechanisms (leakage of the pneumoperitoneum), and does not formally recommend the emptying of the pneumoperitoneum with the trocars still in the operative site^10^, a routine practice advocated by Cavallaro, Piccolo^31^.

## CONCLUSION

The prevalence of incidental gallbladder cancer in cholecystectomies performed between 2018 and 2021 at the Lauro Wanderley University Hospital was 0.16%. Incidental gallbladder cancer is a rare pathology, but not negligible in the routine of the General Surgeon. Its diagnosis, staging, and treatment directly affect patients’ prognosis. Some technical aspects may not always be remembered by surgeons and may interfere with the patient’s subsequent treatment and prognosis.
